# Developing a user-friendly interface for the Lives Saved Tool: LiST Online

**DOI:** 10.7189/jogh.11.03101

**Published:** 2021-09-04

**Authors:** Talata Sawadogo-Lewis, Robert McKinnon, Jill Wyman, William Winfrey, Timothy Roberton

**Affiliations:** 1 Johns Hopkins Bloomberg School of Public Health, Baltimore, Maryland, USA; 2 Avenir Health, Glastonbury, Connecticut, USA

The Lives Saved Tool (LiST) is a mathematical modeling tool currently maintained by the Institute for International Programs (IIP) at the Johns Hopkins Bloomberg School of Public Health. Based on early modelling work for the Lancet Child Survival series 2003, the tool has evolved significantly. In 2008, LiST was integrated as a module in the Spectrum Policy Modeling System (Spectrum) software package – developed and managed by Avenir Health. LiST runs within the Spectrum package, making use of data and calculations from other modules in Spectrum: demographic data from the DemProj module, HIV and AIDS data from the AIM module, and family planning data from the FamPlan module [1]. Additional outcomes were added including interventions that impact stillbirths, birth outcomes and maternal mortality in 2011 [2], and diarrhea and pneumonia incidence were added in 2013 [3]. Stunting and wasting were added in 2008 [4], with additional nutrition outcomes related to the 2012 World Health Assembly (WHA) targets added in 2017 [5]. Today, the most recent version of the tool (v.6.0) can model the impact of 77 interventions along the continuum of care on 28 different causes of maternal, neonatal and under-five death, along with 5 of the 6 WHA nutrition outcomes and stillbirths.

LiST has been used by various agencies, ranging from academic institutions to non-governmental organizations (NGOs) to government agencies, typically for strategic planning, evaluation, or advocacy [6]. However, many groups that have used LiST have required support from the LiST team in the form of training workshops for local teams and/or support for running analyses. Indeed, of 122 peer-reviewed articles published that use LiST (available here), 56 have a named author that is a current or former IIP faculty or staff member. While this list does not capture internal reports or other grey literature, for which LiST may have been used independently, it does suggest that having support from IIP is often useful.

This situation is due in no small part to the fact that although LiST is free to download, the learning curve for how to use the software is perceived by many to be steep [7]. Typically, workshops will last between two and five days depending on how many functionalities are included in the training, as well as the size of the group. Trainings open with a conceptual overview of how LiST works, which is detailed in a publication by Walker [8]. This is followed by hands-on sessions with the software. Additional conceptual training may be added for specific functionalities (eg, subnational wizard, LiST Costing, etc.). By the end of the training sessions, most users are able to navigate the software but are often limited in their ability to carry out more advanced analyses independently.

**Figure:**
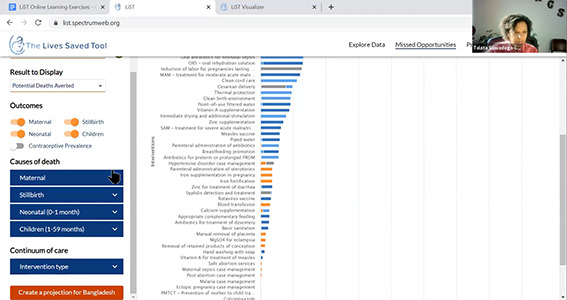
Photo: Using LiST Online for remote training (from the author’s own collection, used with permission).

To improve usability, the LiST team at IIP in partnership with Avenir Health began the process of developing an online version of LiST. Specifically, the LiST team aimed to address the usability challenges by developing a tool that: (1) would reduce friction on users’ end by removing the need to download and install, (2) would increase the focus on LiST features of Spectrum by making LiST itself the entry point to the tool, (3) would have an intuitive “walk-through” approach, guiding the user through the analysis process reducing the need for formal training, (4) would promote collaborative work through a user accounts/library system, and (5) would be browser-based, and therefore be able to be run on all operating systems including macOS.

## DEVELOPMENT PROCESS

**Figure 1:**
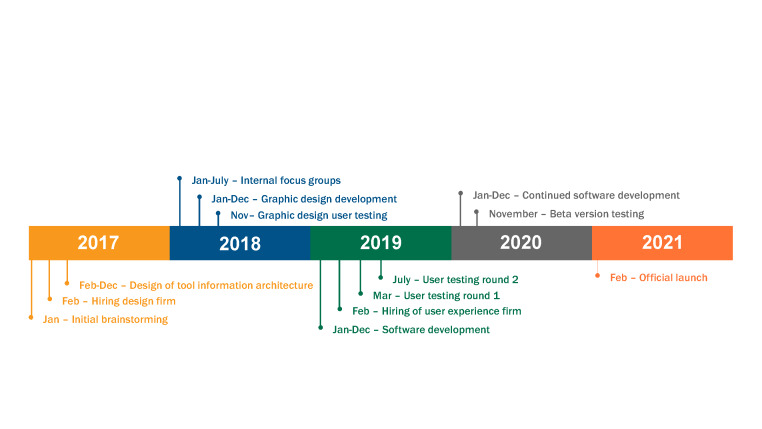
LiST Online development process.

The LiST team began the development process for LiST Online in late 2018 with brainstorming and informal internal focus-group sessions (**Figure 1**). Throughout development the LiST team sought external feedback and guidance from many sources. The LiST team worked with a design firm to guide the aesthetic and user experience of the tool. As part of this process, the external design firm led two rounds of user testing, the findings of which were incorporated in the tool. When a beta version was available, the LiST team held user-testing sessions with different potential user groups, including researchers, students, and public health practitioners. LiST Online was formally launched in January 2021 and is available at https://list.spectrumweb.org.

## KEY FEATURES OF LIST ONLINE

Four features of LiST Online are particularly exciting and highlighted here.

### 1. Identical calculations for LiST Desktop and LiST Online

A key principle that we adopted for LiST Online was that the results it produces should be identical with the desktop version. To enable consistency across versions, Avenir Health reconfigured the desktop version of LiST to run as a server with an application programming interface (API) layer on top to act as the computation engine for LiST Online. Thus, although LiST Online feels different and more streamlined with fewer options presented in the interface, the calculations are in fact the same as the calculations of the desktop version. A further advantage of this setup is that when the calculations of LiST desktop are updated, LiST Online is also automatically updated, meaning that the two versions are always in sync, and users of LiST Online are always using the most up-to-date version.

### 2. Alternative entry points tailored to different needs

**Figure 2:**
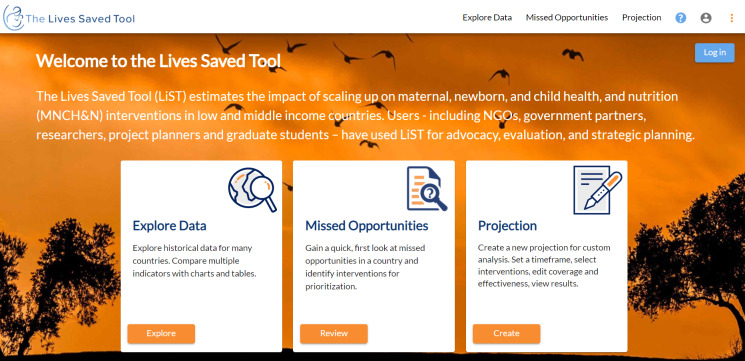
LiST Online entry points.

When people navigate to LiST Online, they see three panels as entry points to LiST (**Figure 2**):

#### 2.1. Explore data

“Explore data” serves as a platform through which users can quickly and easily access the data contained in LiST. These data include mortality, effectiveness, intervention coverage, health, and nutrition, and causes of death. Users can view these data one country at a time through multiple data visualizations and download these data as Excel spreadsheets or PNG files. This functionality allows users to access all of the data in LiST and to visualize these data over time.

#### 2.2. Missed opportunities

“Missed opportunities” enables users to see at a glance which interventions have the highest potential for impact on either mortality or stunting in a country. This can be a useful first step to prioritize among various interventions for reducing child and maternal mortality. Missed Opportunities answers the question “what would happen if the entire population were to reach 90% coverage of each intervention individually in the following year?”. By highlighting it on the home page, we hope to increase its use.

#### 2.3. Projection

The third panel is “Create a projection”, where a user can immediately create a projection of how health outcomes will change as a result of changes in coverage of interventions. Users can also link to the core feature of LiST via the other two panels. For example, if a user is exploring data or missed opportunities, there is an option to create a projection with the country and baseline data they are exploring.

### 3. Walk-through approach

In LiST Online, we have changed the projection creation mechanism to be a “wizard-style” process that walks users through four sequential steps. First, users create a projection by entering basic information including geography, a projection name, primary data source and projection years. Second, they select the interventions they wish to analyze, create scale up patterns and review intervention effectiveness values. In the third step users can review baseline conditions including baseline mortality, cause of death structure, morbidity, nutritional statuses and selected demographic parameters. Users can then examine results covering more than sixty distinct outcomes. In the fourth step, results can be presented in either tabular or graph form and be downloaded as images or Excel spreadsheets for further analysis. This guided “walk-through” process makes using LiST more straightforward, particularly for first-time users.

### 4. User accounts and projection library

A user account is required only if the user wants to save his/her projection. By having an account, a user can return to previously created projections. Projections from a user library can be shared with other users, duplicated, and downloaded. Notes and tags can be added to projections to facilitate searching and organizing. Over time the system of user libraries will become a collaborative space for teams working on a common project, researchers investigating a similar topic and teachers for exposing key elements of epidemiology or public health.

## CONCLUSION

LiST is a public good that has always been freely downloadable. Now it can be used in real time by anyone with an internet connection, regardless of their operating system. With LiST Online, we have created a tool that is easy to use and which can run with minimal external support. It automatically updates data and assumptions and will eventually be able to link to other platforms. We hope this will lead to more people using LiST for evidence-based decision-making at all levels of government and civil society independently – our small contribution towards decolonizing global health.
